# Clinical Profile and Outcome Analysis of Ear-Nose-Throat Symptoms in SARS-CoV-2 Omicron Subvariant Infections

**DOI:** 10.3389/ijph.2023.1606403

**Published:** 2023-10-10

**Authors:** Yixuan Liu, Xiaoling Huang, Peifan Li, Di Wang, Haoning Yin, Na Wang, Yan Luo, Huawei Li, Shan Sun

**Affiliations:** ^1^ Scientific Research Division, Eye & ENT Hospital, Shanghai, China; ^2^ Otolaryngology Research Institute, Eye & ENT Hospital, Shanghai, China; ^3^ NHC Key Laboratory of Hearing Medicine, Fudan University, Shanghai, China; ^4^ Department of Otolaryngology Head and Neck Surgery, The Second Affiliated, Hospital of Anhui Medical University, Hefei, China; ^5^ Clinical Research Unit of Eye & ENT Hospital, Fudan University, Shanghai, China; ^6^ No. 2 High School of East China Normal University, Shanghai, China; ^7^ School of Public Health, Fudan University, Shanghai, China; ^8^ Medical Dean’s Reception Office, Fudan University, Shanghai, China; ^9^ State Key Laboratory of Medical Neurobiology, Fudan University, Shanghai, China

**Keywords:** COVID-19, ENT, network survey, cross-sectional study, symptomatic rate

## Abstract

**Objective:** This study aimed to investigate the clinical characteristics and outcomes of ear-nose-throat (ENT) symptoms in SARS-CoV-2 Omicron infected patients resulting from local transmission.

**Methods:** A convenience sampling network survey was conducted among individuals infected with SARS-CoV-2 to examine the characteristics and progression of ENT symptoms associated with local transmission. The survey comprised 52 questions, and univariable and multivariable logistic regression analyses were employed to assess the rate, severity, and outcome of ENT symptoms across different genders and age groups.

**Results:** Among the 1,366 individuals included in the investigation, a peak in new infections occurred on 20th December, and the majority (78.4%) were female. The most common symptoms reported were coughing (90.6%), nasal congestion (77.2%), and runny nose (74.3%). Otologic symptoms were predominantly represented by tinnitus (29.7%).

**Conclusion:** The rate of specific symptoms showed a significant correlation with age and gender. It is crucial to provide timely medical intervention, especially for female patients. This study offers a comprehensive understanding of the symptom spectrum in individuals infected with the virus, providing valuable insights for the development of targeted symptom management strategies.

## Introduction

The COVID-19 pandemic is a global outbreak of coronavirus, an infectious disease caused by the severe acute respiratory syndrome coronavirus 2 (SARS-CoV-2) virus. As of 12 April 2023, the global impact of COVID-19 has been immense, with over 762 million confirmed cases and nearly 6.9 million deaths [1]. In China, after 3 years of implementing the “dynamic zero” policy, the country’s COVID-19 prevention and control measures have been gradually adjusted. The State Council Joint Prevention and Control Mechanism released the “Notice on Further Optimizing the Scientific and Accurate Prevention and Control of COVID-19” on 11 November 2022, prompting corresponding actions by the Shanghai Municipal Health Commission. Measures such as scaling back large-scale nucleic acid testing (NAT), modifying home isolation strategies, and suspending close contact tracing were promptly implemented. Surveillance data from 25 January 2023, revealed a widespread transmission of SARS-CoV-2 Omicron nationwide, with two sub-variants (BA.5.2 and BF.7) of the Omicron BA.5 variant dominating the current epidemic [2].

Previous studies have indicated that the Omicron BA.5 variant exhibits increased transmissibility and immune evasion capabilities, potentially impacting the host’s immune response and vaccine effectiveness when compared to other variants [3]. However, this variant primarily affects the upper respiratory tract, and the risk of developing pneumonia or severe illness may be lower compared to influenza. Common symptoms of COVID-19 include fever, cough, and fatigue [4], while sore throat and loss of smell are frequently observed in the ear, nose, and throat (ENT) manifestations of the disease [5]. Although fewer cases of hearing loss, tinnitus, vertigo, and related symptoms have been reported in confirmed COVID-19 patients [6, 7], research suggests that these conditions could be potential long-term consequences of the infection, leading to reduced quality of life and negative impacts on communication and daily activities [8–10]. Studies have identified angiotensin-converting enzyme 2 (ACE-2) receptors, which the SARS-CoV-2 virus attaches to, in various regions of the middle and inner ear, including middle ear tissues, the eustachian tube, hair cells in Corti’s organ, vascular striae, and spiral ganglion cells in mouse tissues [11]. Additionally, SARS-CoV-2 viral particles have been detected in the mastoid and middle ear of deceased individuals infected with the virus [12]. Although some individuals infected with COVID-19 do not experience hearing loss, studies have suggested that the infection may have detrimental effects on the outer hair cells in the cochlea [13, 14].

Taste and smell disturbances are common early symptoms of coronavirus infections and often present as initial or solitary symptoms [15–17]. Given the widespread and rapid spread of SARS-CoV-2 infection, it is crucial to comprehensively understand its range of clinical manifestations. However, specific guidelines for managing patients with persistent taste and smell disorders, vertigo, hearing loss, or tinnitus are currently lacking. The treatment of otolaryngological consequences resulting from COVID-19 will continue to be an important issue in the coming years. Further investigation into these symptoms can provide valuable insights for both short-term and long-term management of similar patients.

To elucidate the clinical features and progression of infection, establish a foundation for improving the symptom spectrum of Omicron variant-infected patients, and offer guidance for targeted improvement strategies, we conducted a network survey of individuals infected with SARS-CoV-2 Omicron using convenience sampling.

## Methods

### Study Population

The study enrolled individuals who had been confirmed as infected with SARS-CoV-2 Omicron through nucleic acid amplification testing or rapid antigen testing between 1 December 2022 and 31 January 2023.

To ensure the reliability of the data, certain exclusion criteria were applied. Individuals who were unable to provide truthful information, those who omitted important details or exhibited clear logical errors regarding their gender, age, height, weight, or infection date, and those who submitted duplicate information were excluded from the study. The questionnaire survey was conducted nationwide from 19 January 2023, to 11 February 2023. Ethical considerations were strictly followed, in accordance with the Measures for the Ethical Review of Biomedical Research Involving Humans of the National Health Commission of the People’s Republic of China. The research protocol received ethical approval from the Eye and ENT Hospital Ethics Panel at Fudan University (Approval No. 2022127) for international data collection.

### Primary Outcomes

#### ENT Symptoms

This study aimed to assess the prevalence of various symptoms including hearing loss, tinnitus, stuffiness, earache, vertigo, nasal obstruction, rhinorrhea, sense of smell, cough, sore throat, and reduced sense of taste. Participants were requested to rate the impact of each symptom on their daily life using a scale from 0 (no impact) to 10 (extreme impact) to determine the severity of each symptom. Additionally, participants were asked to report the outcome of each symptom, categorizing it as untreated and self-healed, completely improved after treatment, partially improved after treatment, or residual symptoms. Furthermore, the study examined the medical treatment sought for ENT-related symptoms to evaluate the medical demand associated with the occurrence of these symptoms.

#### Medical History

The study also examined pre-existing medical conditions related to the ear, nose, and throat (ENT) including tinnitus, otitis media, rhinitis, and pharyngitis, prior to the onset of the new coronavirus infection.

### Content and Methods of the Study

The questionnaire was collaboratively developed by two experienced doctors specializing in otolaryngology and public health. It consisted of eight sections, namely: informed consent, basic patient information, date of SARS-CoV-2 infection, ENT symptoms and outcomes, sleep quality, mental health status post-infection, pre-existing ENT medical history, and underlying medical conditions. In total, the questionnaire comprised 52 questions, utilizing formats such as single-choice, multiple-choice, and self-filling. For more detailed content of the questionnaire, please refer to the following link: [18] (Supplementary Material S1).

This study employed a cross-sectional research design and utilized convenience sampling to recruit participants. Data collection was carried out through self-administration of electronic questionnaires. We also made efforts to distribute the survey through various online platforms such as Weibo and WeChat. Within the questionnaire, all self-evaluated items concerning the impact of symptoms on daily life were rated on a scale of 0–10, with higher scores indicating a greater severity of impact.

### Statistical Analysis

The statistical analysis and data visualization were performed using R software version 4.2.2. Metric data that followed a normal distribution were presented as x~±s , while categorical variables were described as frequency (percentage), while categorical variables were reported as frequency (percentage). To compare the two groups, the chi-square test was employed for categorical data, and the Wilcoxon rank-sum test was used for ordinal data. A two-tailed *p*-value of less than 0.05 was considered statistically significant.

## Results

### Basic Information

**FIGURE 1:**
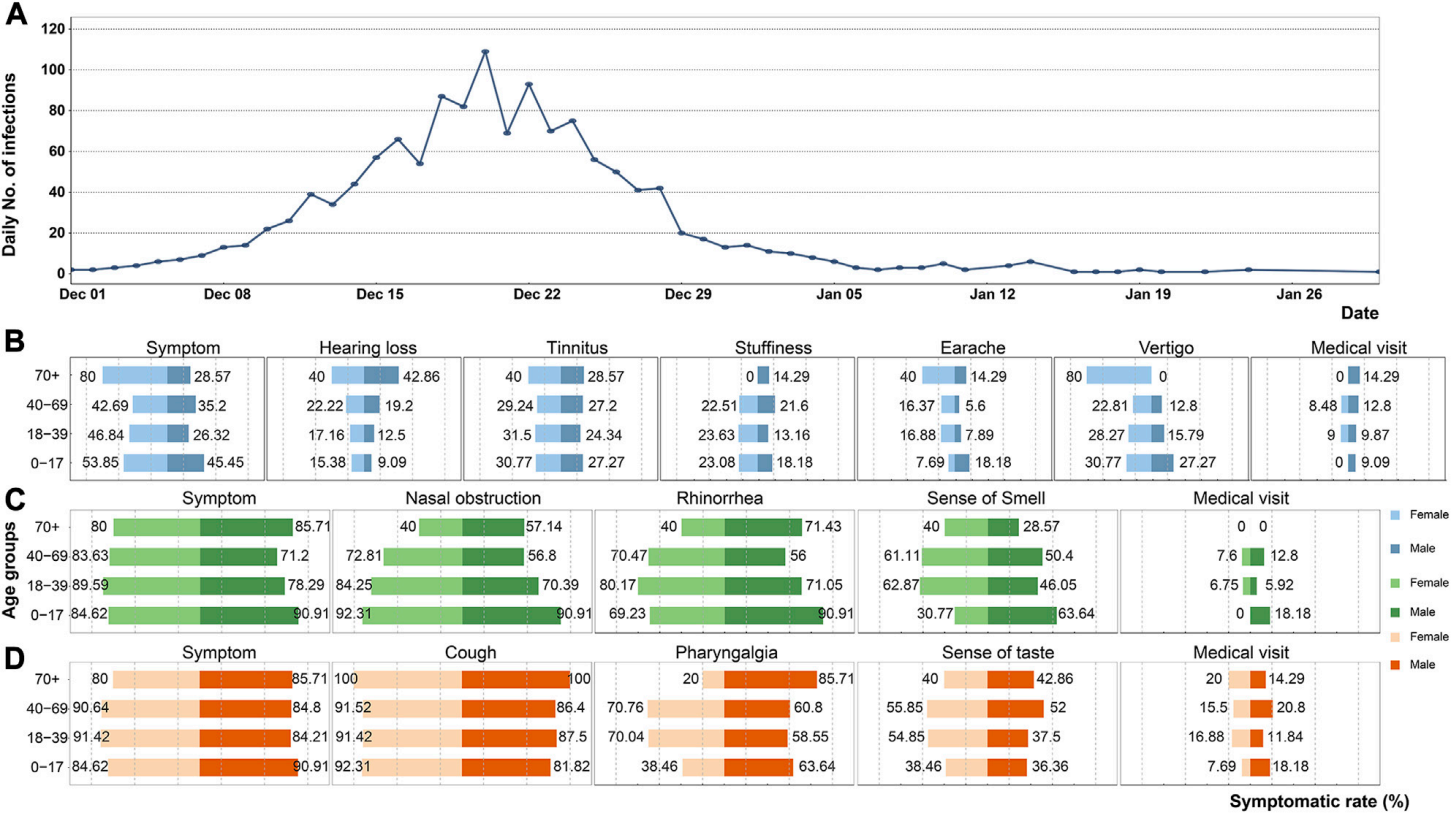
Statistical graphs of related results in the survey (China, 2023). **(A)** Temporal changes of the investigated daily number of infections. **(B)** Symptomatic rate of otologic symptoms stratified by age and gender. **(C)** Symptomatic rate of nasal symptoms stratified by age and gender. **(D)** Symptomatic rate of throat symptoms stratified by age and gender.

A total of 1,366 individuals participated in the questionnaire, out of which 433 (31.7%) resided in Shanghai, and 20 (1.5%) were located outside mainland China. The remaining participants were distributed across various provinces in China. Figure 1A illustrates the trend of newly infected cases investigated, demonstrating a rapid increase since December 1st, with a peak of 109 infections on 20th December. Subsequently, the number fluctuated and declined, with minimal newly identified cases reported after 15th January. It is worth noting that considering the presence of the incubation period and testing delays, the trend of newly infected individuals obtained from the investigation aligns with the changes in the nucleic acid detection rate reported by the CDC. This suggests a relatively high reliability of the investigation [2].

Among the 1,366 infected individuals, 1,071 (78.4%) were female. Common symptoms related to the nose and throat were observed, as the Omicron infection primarily affects the upper respiratory tract. Specifically, 1,054 (77.2%) individuals experienced some degree of nasal obstruction, and 1,015 (74.3%) reported rhinorrhea. In terms of otologic symptoms, tinnitus (29.7%) and vertigo (24.2%) were the most prevalent. It is worth noting that a minority of patients had a history of hypertension or diabetes, while 773 (56.6%) individuals had a history of ENT disease. Additional demographic data and the rates of symptomatic manifestations are provided in Table 1.

**TABLE 1:** Baseline patient information (China, 2023).

Characteristic (*n* = 1,366)	Level	Overall (%)
Gender (%)	Male	295 (21.6)
	Female	1,071 (78.4)
Education level (%)	Elementary school and below	8 (0.6)
	Junior high school	26 (1.9)
	High school	62 (4.5)
	Undergraduate	849 (62.2)
	Master	313 (22.9)
	Doctor	108 (7.9)
Country (%)	Domestic	1,346 (98.5)
	Foreign	20 (1.5)
Province (%)	In Shanghai	433 (31.7)
	Not in Shanghai	933 (68.3)
Ear symptoms (%)	Yes	581 (42.5)
	No	785 (57.5)
Hearing loss (%)	Yes	249 (18.2)
	No	1,117 (81.8)
Tinnitus (%)	Yes	406 (29.7)
	No	960 (70.3)
Stuffiness (%)	Yes	298 (21.8)
	No	1,068 (78.2)
Earache (%)	Yes	201 (14.7)
	No	1,165 (85.3)
Vertigo (%)	Yes	330 (24.2)
	No	1,036 (75.8)
Nose symptoms (%)	Yes	1,162 (85.1)
	No	204 (14.9)
Nasal obstruction (%)	Yes	1,054 (77.2)
	No	312 (22.8)
Rhinorrhea (%)	Yes	1,015 (74.3)
	No	351 (25.7)
Senses (%)	Yes	804 (58.9)
	No	562 (41.1)
Throat symptoms (%)	Yes	1,225 (89.7)
	No	141 (10.3)
Cough (%)	Yes	1,237 (90.6)
	No	129 (9.4)
Pharyngalgia (%)	Yes	924 (67.6)
	No	442 (32.4)
Sense (%)	Yes	717 (52.5)
	No	649 (47.5)
Medical history of ENT (%)	Yes	773 (56.6)
	No	593 (43.4)
Hypertension (%)	Yes	88 (6.4)
	No	1,278 (93.6)
Diabetes (%)	Yes	26 (1.9)
	No	1,340 (98.1)
Age group (%)	0–17	24 (1.8)
	18–39	863 (63.2)
	40–69	467 (34.2)
	70+	12 (0.9)
BMI (%)	Low BMI	133 (9.7)
	Normal	792 (58.0)
	Obese	240 (17.6)
	Obesity	201 (14.7)

### Symptomatic Rate and Outcome of Otologic Symptoms

In terms of otologic symptoms, we observed a higher rate of symptoms among female individuals. This higher rate was particularly notable for symptoms such as stuffiness, earache, and vertigo. Additionally, females appeared to be at a greater risk in terms of the impact of otologic symptoms on their daily lives and the progression of symptoms.

When considering age groups, the rate of hearing loss symptoms increased with age, as shown in Table 2. The rate of tinnitus symptoms, on the other hand, was similar across all age groups, as depicted in Figure 1B. Earache and vertigo were more commonly observed in individuals over the age of 70. Among females in this age group, four individuals (80.0%) reported experiencing vertigo, whereas no males over 70 reported vertigo symptoms, as shown in Figure 1B.

**TABLE 2:** The symptomatic rate, severity and outcome of otologic symptoms among different genders and age groups (China, 2023).

Characteristic		Male (*n* = 295)	Female (*n* = 1,071)	*p*	0–17 (*n* = 24)	18–39 (*n* = 863)	40–69 (*n* = 467)	70+ (*n* = 12)	*p*
**Ear symptoms (%)**	Y	91 (30.8)	490 (45.8)	**<0.001**	12 (50.0)	373 (43.2)	190 (40.7)	6 (50.0)	0.65
	N	204 (69.2)	581 (54.2)		12 (50.0)	490 (56.8)	277 (59.3)	6 (50.0)	
Hearing loss (%)	Y	47 (15.9)	202 (18.9)	0.285	3 (12.5)	141 (16.3)	100 (21.4)	5 (41.7)	**0.017**
	N	248 (84.1)	869 (81.1)		21 (87.5)	722 (83.7)	367 (78.6)	7 (58.3)	
Tinnitus (%)	Y	76 (25.8)	330 (30.8)	0.108	7 (29.2)	261 (30.2)	134 (28.7)	4 (33.3)	0.935
	N	219 (74.2)	741 (69.2)		17 (70.8)	602 (69.8)	333 (71.3)	8 (66.7)	
Stuffiness (%)	Y	50 (16.9)	248 (23.2)	**0.027**	5 (20.8)	188 (21.8)	104 (22.3)	1 (8.3)	0.717
	N	245 (83.1)	823 (76.8)		19 (79.2)	675 (78.2)	363 (77.7)	11 (91.7)	
Earache (%)	Y	22 (7.5)	179 (16.7)	**<0.001**	3 (12.5)	132 (15.3)	63 (13.5)	3 (25.0)	0.594
	N	273 (92.5)	892 (83.3)		21 (87.5)	731 (84.7)	404 (86.5)	9 (75.0)	
Vertigo (%)	Y	43 (14.6)	287 (26.8)	**<0.001**	7 (29.2)	225 (26.1)	94 (20.1)	4 (33.3)	**0.081**
	N	252 (85.4)	784 (73.2)		17 (70.8)	638 (73.9)	373 (79.9)	8 (66.7)	
**Severity of ear symptoms**									
Hearing loss [mean (SD)]		2.01 (2.30)	2.04 (2.16)	0.859	1.75 (2.21)	1.95 (2.12)	2.16 (2.28)	3.42 (3.48)	**0.05**
Tinnitus [mean (SD)]		2.53 (2.75)	2.43 (2.48)	0.559	2.08 (2.08)	2.46 (2.57)	2.43 (2.51)	2.92 (2.84)	0.811
Stuffiness [mean (SD)]		1.92 (2.19)	2.16 (2.36)	0.112	1.54 (1.53)	2.10 (2.30)	2.17 (2.41)	1.75 (1.36)	0.561
Earache [mean (SD)]		1.51 (1.50)	1.85 (1.99)	**0.006**	1.29 (1.23)	1.78 (1.88)	1.78 (1.93)	2.33 (2.71)	0.458
Vertigo [mean (SD)]		1.85 (2.03)	2.43 (2.53)	**<0.001**	2.62 (2.62)	2.35 (2.47)	2.17 (2.30)	3.83 (3.90)	**0.079**
**Outcome of ear symptoms**									
Hearing loss [mean (SD)]		1.52 (1.21)	1.55 (1.20)	0.689	1.25 (0.85)	1.50 (1.15)	1.63 (1.28)	2.42 (1.78)	**0.01**
Tinnitus [mean (SD)]		1.79 (1.43)	1.81 (1.40)	0.827	1.79 (1.41)	1.78 (1.38)	1.83 (1.45)	2.17 (1.59)	0.782
Stuffiness [mean (SD)]		1.36 (0.92)	1.54 (1.13)	**0.01**	1.38 (0.88)	1.49 (1.06)	1.53 (1.17)	1.25 (0.45)	0.701
Earache [mean (SD)]		1.23 (0.74)	1.35 (0.86)	**0.039**	1.17 (0.38)	1.33 (0.84)	1.30 (0.83)	1.83 (1.53)	0.128
Vertigo [mean (SD)]		1.32 (0.80)	1.56 (1.12)	**<0.001**	1.71 (1.23)	1.52 (1.06)	1.46 (1.03)	2.42 (1.68)	**0.014**
**Medical visit**	NA	204 (69.2)	581 (54.2)	**<0.001**	12 (50.0)	490 (56.8)	277 (59.3)	6 (50.0)	0.716
	Y	33 (11.2)	93 (8.7)		1 (4.2)	79 (9.2)	45 (9.6)	1 (8.3)	
	N	58 (19.7)	397 (37.1)		11 (45.8)	294 (34.1)	145 (31.0)	5 (41.7)	

The font of the *p*-value less than 0.05 is bolded.

**FIGURE 2:**
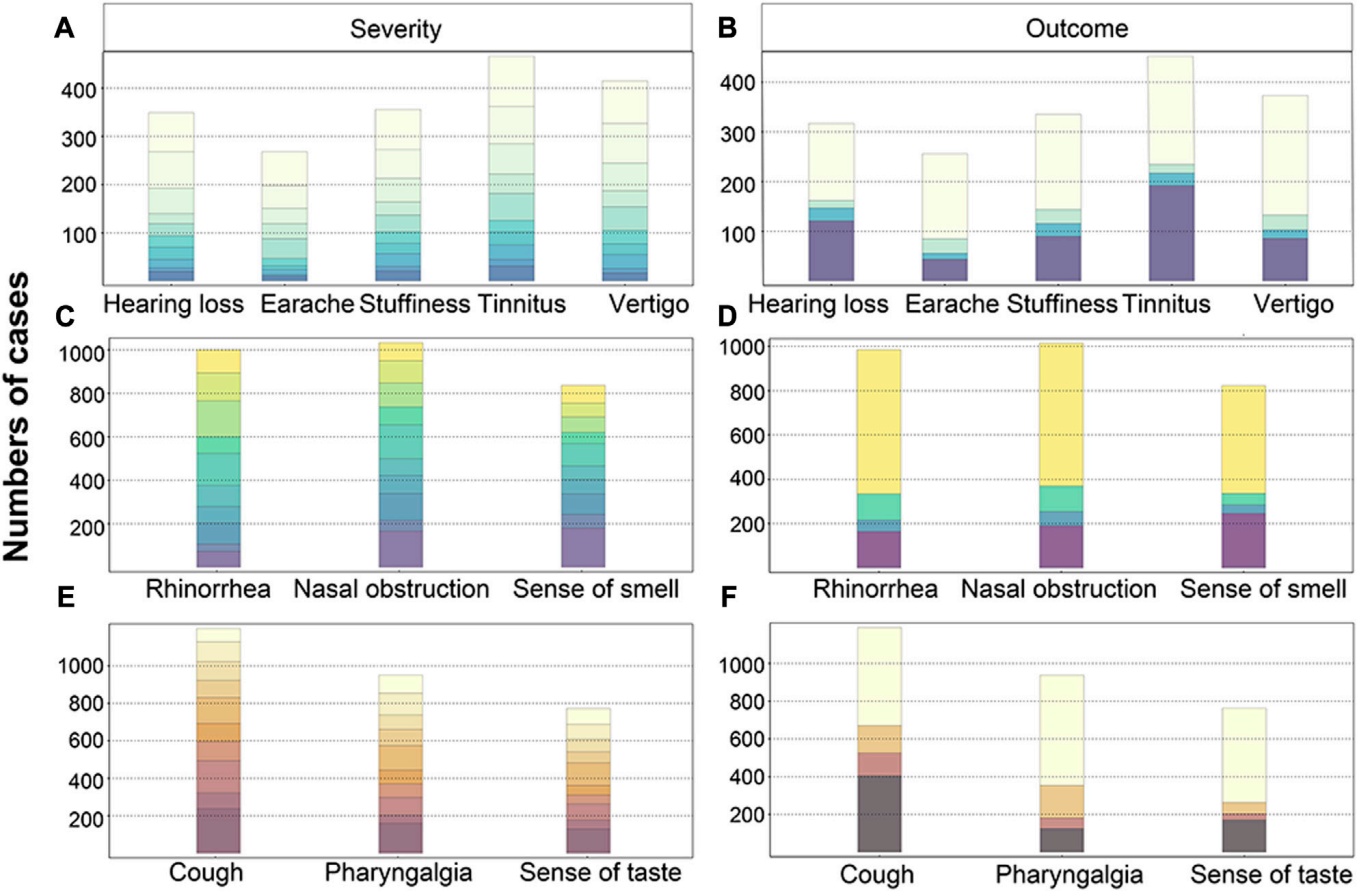
Distribution of severity **(A,C,E)** and outcomes **(B,D,F)** of otolaryngological symptoms . The number of cases with varying scores of severities for ENT symptoms, including the otologic **(A)**, nasal **(C)**, and throat **(E)** symptoms. The number of cases with differing outcomes for otologic **(B)**, nasal **(D)**, and throat **(F)** symptoms. Note: The scores are represented by different colors, ranging from 1 to 10. Lighter shades indicate lower scores, while darker shades indicate higher scores. The vertical axis represents the number of cases (China, 2023).

Apart from the symptomatic rate, similar patterns were observed in terms of the impact on daily life and symptom progression. Overall, the impact of otologic symptoms on patients’ lives was relatively minor, but tinnitus had a more common and severe impact compared to other symptoms, as indicated in Table 2. Furthermore, earache and vertigo had a greater impact on females. A few patients sought medical attention after experiencing otologic symptoms related to COVID-19. In most cases, these symptoms improved or partially improved after treatment, while tinnitus and hearing loss were common residual symptoms, as depicted in Figures 2A, B.

The impact of BMI on otologic symptoms was found to be insignificant, with only a higher rate of ear pain observed in underweight individuals, as shown in Supplementary Table S1.

### Symptomatic Rate and Outcome of Nasal Symptoms

Nasal obstruction, rhinorrhea, and loss of smell had a significant impact on the daily lives of patients, as indicated in Table 3. Nearly half of the patients rated the impact of nasal symptoms as moderate to severe, with scores ranging from 4 to 10, as shown in Figure 2C. The symptomatic rate of rhinologic symptoms was generally higher among female cases, and these symptoms had a more severe impact and progression. Nasal obstruction, rhinorrhea, and other nasal symptoms were observed across all age groups, with a higher symptomatic rate among younger individuals. In fact, the symptomatic rate of nasal obstruction in children exceeded 90%, as depicted in Figure 1C. However, the impact and progression of symptoms were less severe compared to the middle-aged group.

**TABLE 3:** The symptomatic rate, severity and outcome of nasal symptoms among different genders and age groups (China, 2023).

Characteristic	Level	Male (*n* = 295)	Female (*n* = 1,071)	*p*	0–17 (*n* = 24)	18–39 (*n* = 863)	40–69 (*n* = 467)	70+ (*n* = 12)	*p*
**Nasal symptoms (%)**	Y	224 (75.9)	938 (87.6)	**<0.001**	21 (87.5)	756 (87.6)	375 (80.3)	10 (83.3)	**0.005**
	N	71 (24.1)	133 (12.4)		3 (12.5)	107 (12.4)	92 (19.7)	2 (16.7)	
Nasal obstruction (%)	Y	192 (65.1)	862 (80.5)	**<0.001**	22 (91.7)	706 (81.8)	320 (68.5)	6 (50.0)	**<0.001**
	N	103 (34.9)	209 (19.5)		2 (8.3)	157 (18.2)	147 (31.5)	6 (50.0)	
Rhinorrhea (%)	Y	193 (65.4)	822 (76.8)	**<0.001**	19 (79.2)	678 (78.6)	311 (66.6)	7 (58.3)	**<0.001**
	N	102 (34.6)	249 (23.2)		5 (20.8)	185 (21.4)	156 (33.4)	5 (41.7)	
Senses (%)	Y	142 (48.1)	662 (61.8)	**<0.001**	11 (45.8)	517 (59.9)	272 (58.2)	4 (33.3)	0.146
	N	153 (51.9)	409 (38.2)		13 (54.2)	346 (40.1)	195 (41.8)	8 (66.7)	
**Severity of nasal symptoms**									
Nasal obstruction [mean (SD)]		4.42 (3.33)	5.03 (3.22)	**0.004**	4.71 (2.82)	5.17 (3.23)	4.45 (3.27)	3.00 (2.80)	**<0.001**
Rhinorrhea [mean (SD)]		4.24 (3.12)	4.83 (3.14)	**0.004**	4.58 (2.90)	4.96 (3.15)	4.28 (3.10)	3.25 (2.93)	**0.001**
Senses [mean (SD)]		3.71 (3.29)	4.21 (3.40)	**0.025**	3.25 (3.12)	4.15 (3.38)	4.07 (3.38)	3.42 (3.94)	0.52
**Outcome of nasal symptoms**									
Nasal obstruction [mean (SD)]		2.07 (1.24)	2.41 (1.30)	**<0.001**	2.79 (1.35)	2.42 (1.30)	2.17 (1.26)	2.00 (1.35)	**0.002**
Rhinorrhea [mean (SD)]		2.03 (1.18)	2.30 (1.25)	**0.001**	2.50 (1.32)	2.31 (1.25)	2.12 (1.20)	1.92 (1.38)	**0.039**
Senses [mean (SD)]		2.03 (1.38)	2.29 (1.47)	**0.006**	2.04 (1.37)	2.21 (1.42)	2.31 (1.52)	1.83 (1.53)	0.375
**Medical visit**	NA	71 (24.1)	133 (12.4)	**<0.001**	3 (12.5)	107 (12.4)	92 (19.7)	2 (16.7)	**0.006**
	Y	27 (9.2)	74 (6.9)		2 (8.3)	57 (6.6)	42 (9.0)	0 (0.0)	
	N	197 (66.8)	864 (80.7)		19 (79.2)	699 (81.0)	333 (71.3)	10 (83.3)	

The font of the *p*-value less than 0.05 is bolded.

Few patients sought treatment specifically for rhinologic symptoms. The results indicated a low short-term spontaneous recovery rate for the loss of sense of smell, but few patients reported residual symptoms, as shown in Figures 2C, D. The impact of BMI on rhinologic symptoms was found to be insignificant, as demonstrated in Supplementary Table S2. However, there was a trend of more severe progression of nasal obstruction in underweight individuals.

### Symptomatic Rate and Outcome of Throat Symptoms

Throat symptoms were highly prevalent among individuals infected with SARS-CoV-2, with rates exceeding 80%. Similar to ear and nose symptoms, female individuals exhibited higher rates of symptomatic presentations, while no clear association was observed between age (Table 4) or BMI (Supplementary Table S3) and symptomatic rates.

**TABLE 4:** The symptomatic rate, severity and outcome of throat symptoms among different genders and age groups (China, 2023).

Characteristic	Level	Male (*n* = 295)	Female (*n* = 1,071)	*p*	0–17 (*n* = 24)	18–39 (*n* = 863)	40–69 (*n* = 467)	70+ (*n* = 12)	*p*
***n***		295	1,071		24	863	467	12	
**Throat symptoms (%)**	Y	250 (84.7)	975 (91.0)	**0.002**	21 (87.5)	778 (90.2)	416 (89.1)	10 (83.3)	0.793
	N	45 (15.3)	96 (9.0)		3 (12.5)	85 (9.8)	51 (10.9)	2 (16.7)	
Cough (%)	Y	257 (87.1)	980 (91.5)	**0.03**	21 (87.5)	783 (90.7)	421 (90.1)	12 (100.0)	0.652
	N	38 (12.9)	91 (8.5)		3 (12.5)	80 (9.3)	46 (9.9)	0 (0.0)	
Pharyngalgia (%)	Y	178 (60.3)	746 (69.7)	**0.003**	12 (50.0)	587 (68.0)	318 (68.1)	7 (58.3)	0.263
	N	117 (39.7)	325 (30.3)		12 (50.0)	276 (32.0)	149 (31.9)	5 (41.7)	
Sense (%)	Y	129 (43.7)	588 (54.9)	**0.001**	9 (37.5)	447 (51.8)	256 (54.8)	5 (41.7)	0.272
	N	166 (56.3)	483 (45.1)		15 (62.5)	416 (48.2)	211 (45.2)	7 (58.3)	
**Severity of throat symptoms**									
Cough [mean (SD)]		5.46 (3.33)	5.78 (3.19)	0.131	5.46 (2.98)	5.78 (3.21)	5.60 (3.29)	5.58 (2.39)	0.783
Pharyngalgia [mean (SD)]		4.05 (3.13)	4.52 (3.24)	**0.028**	3.50 (3.02)	4.51 (3.26)	4.32 (3.17)	3.25 (2.56)	0.197
Sense [mean (SD)]		3.66 (3.25)	3.87 (3.28)	0.329	3.04 (3.03)	3.77 (3.26)	3.99 (3.31)	3.25 (3.19)	0.375
**Outcome of throat symptoms**									
Cough [mean (SD)]		2.74 (1.46)	3.13 (1.47)	**<0.001**	2.83 (1.46)	3.03 (1.46)	3.07 (1.50)	3.58 (1.44)	0.513
Pharyngalgia [mean (SD)]		1.99 (1.09)	2.22 (1.20)	**0.003**	1.58 (0.72)	2.17 (1.17)	2.21 (1.23)	2.00 (0.95)	**0.087**
Sense [mean (SD)]		1.87 (1.25)	2.07 (1.32)	**0.024**	1.62 (0.97)	2.00 (1.28)	2.09 (1.36)	1.92 (1.51)	0.297
**Medical visit**	NA	45 (15.3)	96 (9.0)	**0.007**	3 (12.5)	85 (9.8)	51 (10.9)	2 (16.7)	0.956
	Y	47 (15.9)	175 (16.3)		3 (12.5)	138 (16.0)	79 (16.9)	2 (16.7)	
	N	203 (68.8)	800 (74.7)		18 (75.0)	640 (74.2)	337 (72.2)	8 (66.7)	

The font of the *p*-value less than 0.05 is bolded.

Among the throat symptoms, cough was the most common, with a symptomatic rate of up to 100% in elderly patients, as shown in Figure 1D. Nearly 600 patients rated the impact of cough as severe, with scores ranging from 7 to 10. Furthermore, over 400 patients reported cough as a post-acute sequela of COVID-19 (Figures 2E, F). Additionally, females experienced more severe throat pain and poorer outcomes for all throat symptoms compared to males, as indicated in Table 4. Throat symptoms also exhibited higher rates of medical visits compared to ear or nose symptoms, as depicted in Figure 1.

## Discussion

SARS-CoV-2 Omicron infection typically presents with mild or subclinical features, primarily manifesting as symptoms like dry cough, fever, and diarrhea. However, as the pandemic has progressed, neurological manifestations have also been observed in some individuals. The range of clinical presentations associated with SARS-CoV-2 infection is expanding, with reported cases of Guillain-Barre syndrome as a separate entity [19–21]. While research on COVID-19 symptoms has primarily focused on the respiratory system, such as pulmonary symptoms [22], here is relatively limited research on ENT (ear, nose, and throat) aspects. Some studies have reported on hearing and vestibular function [7], including hearing loss [13, 23, 24], tinnitus [25, 26], and vertigo [26, 27]. In comparison to other COVID-19 symptom studies, our research specifically focused on comparing ear, nasal, and throat aspects and utilized a large and representative sample size through cross-sectional survey methods.

Our findings indicate that the symptomatic rate of nasal and throat symptoms is significantly higher than that of ear symptoms. There is a notable correlation between age and the symptomatic rate of certain symptoms. Older adults are less likely to experience nasal obstruction, rhinorrhea, loss of sense of smell, pharyngalgia, and loss of sense of taste. These findings are supported by previous studies [27, 28], that suggest younger individuals are more prone to experiencing ENT symptoms. For instance, a study by Lechien et al. involving 1420 patients found differences in the symptomatic rate of COVID-19 symptoms based on gender and age, with younger individuals more frequently experiencing nasal congestion, rhinorrhea, and pharyngalgia [29]. Another study by Vaira et al. [30] discovered that loss of sense of smell and/or taste commonly occur in the early stages of the disease and gradually disappear, indicating potential viral interference or local inflammation of taste and olfactory receptors rather than permanent neuronal damage or invasion of the central nervous system [30]. Further research is needed to explore the specific mechanisms underlying taste and olfactory disturbances caused by SARS-CoV-2.

Previous reports suggest that older adults tend to have more severe clinical symptoms and outcomes after SARS-CoV-2 Omicron infection [31]. Our research aligns with this understanding, indicating that comorbidities in older individuals may contribute to the increased severity. By comparing and analyzing survey results with existing research [6, 28, 32–37], we have provided new insights and discoveries regarding the symptomatic rate and outcomes of otolaryngological symptoms. We particularly emphasized the importance of addressing tinnitus and hearing loss as symptoms requiring attention and resolution. Additionally, we highlighted the need for greater attention and treatment of otolaryngological symptoms in females, given their higher symptomatic rates and more severe clinical manifestations. Considering the high symptomatic rate of ENT symptoms and their significant impact on quality of life, along with the potential for long-lasting residual effects, it is crucial to prioritize the recognition and management of ENT symptoms associated with SARS-CoV-2 infection.

It’s important to acknowledge the limitations of our study, which utilized cross-sectional internet-based surveys. These surveys are subject to limitations such as sampling biases, as they primarily reach internet audiences, who are typically younger and more educated. Older adults may be less likely to participate in online questionnaires, while women tend to have higher participation rates in online surveys. As our survey specifically targeted SARS-CoV-2 Omicron infected individuals with ENT symptoms, there may be sample biases. Additionally, reports from infected individuals can be influenced by subjective factors, leading to reporting biases. Furthermore, cross-sectional surveys conducted online can only establish correlations between variables and cannot determine causal relationships, resulting in lower levels of evidence.

Given the potential for tinnitus and hearing loss to persist as long-term residual symptoms, healthcare professionals should be vigilant in informing COVID-19 patients about these potential complications to enable early detection and intervention. Moreover, due to the higher symptomatic rate and more severe clinical manifestations of ENT symptoms in female patients, enhanced symptom monitoring and optimized treatment plans are necessary. Lastly, although the elderly population may exhibit a lower symptomatic rate of ENT symptoms, they experience a greater impact on their quality of life and poorer outcomes. Therefore, tailored symptom management strategies for this age group are crucial.

### Conclusion

Our investigation reveals an outbreak of COVID-19 infections in December 2022. We found that otologic symptoms have a relatively minor impact on patients’ lives. However, tinnitus and hearing loss are the most commonly reported residual symptoms. Rhinological symptoms have a relatively high symptomatic rate, but they typically do not persist as residual symptoms. On the other hand, laryngeal symptoms have the highest symptomatic rate and exert the greatest impact on patients’ quality of life. Furthermore, our study highlights that female patients are more vulnerable to these effects. These findings serve as a foundation for enhancing our understanding of the symptom spectrum in patients infected with the Omicron variant and provide valuable insights for targeted symptom management strategies.

## Data Availability

The datasets generated during or analyzed during the current study are available from the corresponding author on reasonable request.
