# Supplementary material for: Clinical Profile and Outcome Analysis of Ear-Nose-Throat Symptoms in SARS-CoV-2 Omicron Subvariant Infections
Source: Int J Public Health. 2023 Oct 10;68:1606403. doi: 10.3389/ijph.2023.1606403 (PMC10619212; doi:10.3389/ijph.2023.1606403)
Supplement: Supplementary file 1 [file Table1.DOCX]

**Supplemental Table S1. The symptomatic rate, severity and outcome of otologic symptoms in different BMI groups.**

| **Characteristic** |  | **Underweight (n = 133)** | **Normal (n = 792)** | **Overweight (n = 240)** | **Obese (n = 201)** | ***p*** |
| --- | --- | --- | --- | --- | --- | --- |
| **Ear symptoms (%)** | Y | 57(42.9) | 345(43.6) | 94(39.2) | 85(42.3) | 0.69 |
|  | N | 76(57.1) | 447(56.4) | 146(60.8) | 116(57.7) |  |
| Hearing loss (%) | Y | 18(13.5) | 152(19.2) | 43(17.9) | 36(17.9) | 0.477 |
|  | N | 115(86.5) | 640(80.8) | 197(82.1) | 165(82.1) |  |
| Tinnitus (%) | Y | 37(27.8) | 225(28.4) | 73(30.4) | 71(35.3) | 0.266 |
|  | N | 96(72.2) | 567(71.6) | 167(69.6) | 130(64.7) |  |
| Stuffiness (%) | Y | 27(20.3) | 174(22.0) | 47(19.6) | 50(24.9) | 0.574 |
|  | N | 106(79.7) | 618(78.0) | 193(80.4) | 151(75.1) |  |
| Earache (%) | Y | 30(22.6) | 117(14.8) | 27(11.2) | 27(13.4) | **0.028** |
|  | N | 103(77.4) | 675(85.2) | 213(88.8) | 174(86.6) |  |
| Vertigo (%) | Y | 35(26.3) | 189(23.9) | 48(20.0) | 58(28.9) | 0.167 |
|  | N | 98(73.7) | 603(76.1) | 192(80.0) | 143(71.1) |  |
| **Severity of ear symptoms** |  |  |  |  |  |  |
| Hearing loss (mean (SD)) |  | 1.92(2.06) | 2.06(2.24) | 1.99(2.12) | 2.05(2.20) | 0.897 |
| Tinnitus (mean (SD)) |  | 2.53(2.56) | 2.36(2.49) | 2.32(2.26) | 2.91(2.96) | 0.038 |
| Stuffiness (mean (SD)) |  | 1.97(2.11) | 2.13(2.35) | 2.00(2.31) | 2.23(2.38) | 0.642 |
| Earache (mean (SD)) |  | 2.14(2.08) | 1.76(1.87) | 1.65(1.86) | 1.76(1.92) | 0.118 |
| Vertigo (mean (SD)) |  | 2.45(2.49) | 2.30(2.42) | 2.07(2.22) | 2.50(2.72) | 0.252 |
| **Outcome of ear symptoms** |  |  |  |  |  |  |
| Hearing loss (mean (SD)) |  | 1.43(1.09) | 1.55(1.20) | 1.58(1.26) | 1.58(1.23) | 0.642 |
| Tinnitus (mean (SD)) |  | 1.85(1.45) | 1.74(1.37) | 1.81(1.39) | 2.01(1.54) | **0.108** |
| Stuffiness (mean (SD)) |  | 1.52(1.13) | 1.49(1.08) | 1.47(1.08) | 1.56(1.14) | 0.817 |
| Earache (mean (SD)) |  | 1.41(0.86) | 1.34(0.89) | 1.28(0.79) | 1.25(0.65) | 0.322 |
| Vertigo (mean (SD)) |  | 1.59(1.16) | 1.51(1.07) | 1.43(0.94) | 1.57(1.09) | 0.402 |
| **Medical visit** | NA | 76(57.1) | 447(56.4) | 146(60.8) | 116(57.7) | 0.219 |
|  | Y | 10(7.5) | 73(9.2) | 16(6.7) | 27(13.4) |  |
|  | N | 47(35.3) | 272(34.3) | 78(32.5) | 58(28.9) |  |

**Supplemental Table S2. The symptomatic rate, severity and outcome of nasal symptoms in different BMI groups.**

| **Characteristic** | **Level** | **Underweight (n = 133)** | **Normal (n = 792)** | **Overweight (n = 240)** | **Obese (n = 201)** | ***p*** |
| --- | --- | --- | --- | --- | --- | --- |
| **Nasal symptoms (%)** | Y | 120(90.2) | 670(84.6) | 199(82.9) | 173(86.1) | 0.266 |
|  | N | 13(9.8) | 122(15.4) | 41(17.1) | 28(13.9) |  |
| Nasal obstruction (%) | Y | 108(81.2) | 608(76.8) | 175(72.9) | 163(81.1) | 0.137 |
|  | N | 25(18.8) | 184(23.2) | 65(27.1) | 38(18.9) |  |
| Rhinorrhea (%) | Y | 102(76.7) | 598(75.5) | 172(71.7) | 143(71.1) | 0.404 |
|  | N | 31(23.3) | 194(24.5) | 68(28.3) | 58(28.9) |  |
| Senses (%) | Y | 84(63.2) | 453(57.2) | 145(60.4) | 122(60.7) | 0.486 |
|  | N | 49(36.8) | 339(42.8) | 95(39.6) | 79(39.3) |  |
| **Severity of nasal symptoms** |  |  |  |  |  |  |
| Nasal obstruction (mean (SD)) |  | 5.11(3.25) | 4.92(3.26) | 4.62(3.32) | 5.02(3.17) | 0.452 |
| Rhinorrhea (mean (SD)) |  | 4.89(3.27) | 4.73(3.10) | 4.66(3.27) | 4.52(3.09) | 0.742 |
| Senses (mean (SD)) |  | 4.36(3.50) | 4.02(3.37) | 4.05(3.36) | 4.30(3.39) | 0.581 |
| **Outcome of nasal symptoms** |  |  |  |  |  |  |
| Nasal obstruction (mean (SD)) |  | 2.66(1.38) | 2.29(1.25) | 2.28(1.33) | 2.37(1.31) | **0.018** |
| Rhinorrhea (mean (SD)) |  | 2.40(1.35) | 2.23(1.21) | 2.25(1.30) | 2.21(1.22) | 0.496 |
| Senses (mean (SD)) |  | 2.39(1.51) | 2.18(1.43) | 2.28(1.50) | 2.31(1.45) | 0.331 |
| **Medical visit** | NA | 13(9.8) | 122(15.4) | 41(17.1) | 28(13.9) | 0.171 |
|  | Y | 7(5.3) | 55(6.9) | 17(7.1) | 22(10.9) |  |
|  | N | 113(85.0) | 615(77.7) | 182(75.8) | 151(75.1) |  |

**Supplemental Table S3. The symptomatic rate, severity and outcome of throat symptoms in different BMI groups.**

| **Characteristic** | **Level** | **Male**  **(n = 295)** | **Female**  **(n = 1071)** | ***p*** | **0-17**  **(n = 24)** | **18-39 (n = 863)** | **40-69 (n = 467)** | **70+ (n = 12)** | ***p*** | **Underweight (n = 133)** | **Normal (n = 792)** | **Overweight (n = 240)** | **Obese (n = 201)** | ***p*** |
| --- | --- | --- | --- | --- | --- | --- | --- | --- | --- | --- | --- | --- | --- | --- |
| **Throat symptoms (%)** | Y | 250(84.7) | 975(91.0) | **0.002** | 21(87.5) | 778(90.2) | 416(89.1) | 10(83.3) | 0.793 | 115(86.5) | 720(90.9) | 214(89.2) | 176(87.6) | 0.282 |
|  | N | 45(15.3) | 96(9.0) |  | 3(12.5) | 85(9.8) | 51(10.9) | 2(16.7) |  | 18(13.5) | 72(9.1) | 26(10.8) | 25(12.4) |  |
| Cough (%) | Y | 257(87.1) | 980(91.5) | **0.03** | 21(87.5) | 783(90.7) | 421(90.1) | 12(100.0) | 0.652 | 113(85.0) | 727(91.8) | 218(90.8) | 179(89.1) | **0.077** |
|  | N | 38(12.9) | 91(8.5) |  | 3(12.5) | 80(9.3) | 46(9.9) | 0(0.0) |  | 20(15.0) | 65(8.2) | 22(9.2) | 22(10.9) |  |
| Pharyngalgia (%) | Y | 178(60.3) | 746(69.7) | **0.003** | 12(50.0) | 587(68.0) | 318(68.1) | 7(58.3) | 0.263 | 92(69.2) | 536(67.7) | 158(65.8) | 138(68.7) | 0.897 |
|  | N | 117(39.7) | 325(30.3) |  | 12(50.0) | 276(32.0) | 149(31.9) | 5(41.7) |  | 41(30.8) | 256(32.3) | 82(34.2) | 63(31.3) |  |
| Sense (%) | Y | 129(43.7) | 588(54.9) | **0.001** | 9(37.5) | 447(51.8) | 256(54.8) | 5(41.7) | 0.272 | 77(57.9) | 399(50.4) | 130(54.2) | 111(55.2) | 0.279 |
|  | N | 166(56.3) | 483(45.1) |  | 15(62.5) | 416(48.2) | 211(45.2) | 7(58.3) |  | 56(42.1) | 393(49.6) | 110(45.8) | 90(44.8) |  |
| **Severity of throat symptoms** |  |  |  |  |  |  |  |  |  |  |  |  |  |  |
| Cough (mean (SD)) |  | 5.46(3.33) | 5.78(3.19) | 0.131 | 5.46(2.98) | 5.78(3.21) | 5.60(3.29) | 5.58(2.39) | 0.783 | 5.48(3.18) | 5.89(3.23) | 5.38(3.17) | 5.58(3.28) | 0.119 |
| Pharyngalgia (mean (SD)) |  | 4.05(3.13) | 4.52(3.24) | **0.028** | 3.50(3.02) | 4.51(3.26) | 4.32(3.17) | 3.25(2.56) | 0.197 | 4.53(3.25) | 4.47(3.24) | 4.16(3.15) | 4.44(3.23) | 0.599 |
| Sense (mean (SD)) |  | 3.66(3.25) | 3.87(3.28) | 0.329 | 3.04(3.03) | 3.77(3.26) | 3.99(3.31) | 3.25(3.19) | 0.375 | 3.89(3.23) | 3.74(3.26) | 3.76(3.21) | 4.18(3.43) | 0.38 |
| **Outcome of throat symptoms** |  |  |  |  |  |  |  |  |  |  |  |  |  |  |
| Cough (mean (SD)) |  | 2.74(1.46) | 3.13(1.47) | **< 0.001** | 2.83(1.46) | 3.03(1.46) | 3.07(1.50) | 3.58(1.44) | 0.513 | 3.07(1.49) | 3.06(1.48) | 3.01(1.44) | 3.01(1.50) | 0.965 |
| Pharyngalgia (mean (SD)) |  | 1.99(1.09) | 2.22(1.20) | **0.003** | 1.58(0.72) | 2.17(1.17) | 2.21(1.23) | 2.00(0.95) | **0.087** | 2.18(1.19) | 2.19(1.20) | 2.12(1.16) | 2.14(1.15) | 0.849 |
| Sense (mean (SD)) |  | 1.87(1.25) | 2.07(1.32) | **0.024** | 1.62(0.97) | 2.00(1.28) | 2.09(1.36) | 1.92(1.51) | 0.297 | 2.17(1.37) | 1.97(1.27) | 2.08(1.39) | 2.10(1.30) | 0.242 |
| **Medical visit** | NA | 45(15.3) | 96(9.0) | **0.007** | 3(12.5) | 85(9.8) | 51(10.9) | 2(16.7) | 0.956 | 18(13.5) | 72(9.1) | 26(10.8) | 25(12.4) | 0.409 |
|  | Y | 47(15.9) | 175(16.3) |  | 3(12.5) | 138(16.0) | 79(16.9) | 2(16.7) |  | 21(15.8) | 124(15.7) | 38(15.8) | 39(19.4) |  |
|  | N | 203(68.8) | 800(74.7) |  | 18(75.0) | 640(74.2) | 337(72.2) | 8(66.7) |  | 94(70.7) | 596(75.3) | 176(73.3) | 137(68.2) |  |
