# Supplementary material for: Clinical Profile and Outcome Analysis of Ear-Nose-Throat Symptoms in SARS-CoV-2 Omicron Subvariant Infections
Source: Int J Public Health. 2023 Oct 10;68:1606403. doi: 10.3389/ijph.2023.1606403 (PMC10619212; doi:10.3389/ijph.2023.1606403)
Supplement: Supplementary file 2 [file DataSheet1.docx]

**Questionnaire on Tinnitus** **(Translated from Chinese)**

1. Name:________
2. Sex:□Male □Female
3. Age:________
4. Hight (cm):________
5. Weight (kg):________
6. Education level:

□Primary school or below

□Junior high school

□Technical secondary school/senior high school

□Junior college/undergraduate degree

□Master's degree

□Doctoral degree

1. Your current occupation:________
2. Location (if outside of China, please specify the country and city)：

□Within China □Outside of China

1. City of residence:________
2. Phone number (optional, can be used for follow-up by the professional team at Fudan University Affiliated Eye, Ear, Nose and Throat Hospital):________
3. Date of COVID-19 infection (based on the earliest positive nucleic acid/antigen test result)

**Ear Symptoms:**

1. Did you experience any ear-related symptoms after being infected with COVID-19 (e.g. hearing loss, tinnitus, stuffy ears, ear pain, dizziness, etc.)?

□Yes □No

1. Did you experience hearing loss after being infected with COVID-19?

□Yes □No

1. Where did you experience the hearing loss after being infected with COVID-19?

□Left ear □Right ear □Both ears

1. Did you experience tinnitus after being infected with COVID-19?

□Yes □No

1. Where did you experience the tinnitus after being infected with COVID-19?

□Left ear □Right ear □Both ears

1. Did the tinnitus affect your attention, work, ability to hear others, or enjoyment of life?

□Yes □Sometimes □No

1. Did the tinnitus make you feel angry, irritable, depressed, anxious, insecure, or strain your relationships with family and friends?

□Yes □Sometimes □No

1. Did the tinnitus make you feel hopeless, out of control, intolerable, or make you think you have a serious illness?

□Yes □Sometimes □No

1. Did the tinnitus affect your sleep? (e.g. difficulty falling asleep, waking up at night, early awakening, etc.)

□No effect □Slight impact □Significant impact □Severe impact or no sleep

1. Were you satisfied with your quality of overall sleep?

□Satisfied □Slightly dissatisfied

□Significantly dissatisfied □Very dissatisfied or couldn't sleep

1. Did you experience ear fullness after being infected with COVID-19?

□Yes □No

1. Where did you experience ear fullness after COVID-19 infection:

□Left ear □Right ear □Both ears

1. Did you experience ear pain after being infected with COVID-19?

□Yes □No

1. Where did you experience ear pain after COVID-19 infection:

□Left ear □Right ear □Both ears

1. Did you experience dizziness (vertigo) after being infected with COVID-19?

□Yes □No

1. Did you see a doctor because of the ear symptoms after COVID-19 infection?

□Yes □No

1. Level of impact on daily life due to ear symptoms after COVID-19 infection (0 means no impact, 10 means extremely impactful) - Hearing loss

□1 □2 □3 □4 □5 □6 □7 □8 □9 □10

1. Level of impact on daily life due to ear symptoms after COVID-19 infection (0 means no impact, 10 means extremely impactful) - Tinnitus

□1 □2 □3 □4 □5 □6 □7 □8 □9 □10

1. Level of impact on daily life due to ear symptoms after COVID-19 infection (0 means no impact, 10 means extremely impactful) - Ear fullness

□1 □2 □3 □4 □5 □6 □7 □8 □9 □10

1. Level of impact on daily life due to ear symptoms after COVID-19 infection (0 means no impact, 10 means extremely impactful) - Ear pain

□1 □2 □3 □4 □5 □6 □7 □8 □9 □10

1. Level of impact on daily life due to ear symptoms after COVID-19 infection (0 means no impact, 10 means extremely impactful) - Dizziness

□1 □2 □3 □4 □5 □6 □7 □8 □9 □10

1. Outcome of ear symptoms after COVID-19 infection - Hearing loss

□None □Completely disappeared after treatment

□Partially disappeared after treatment □Self-healing residual symptoms

1. Outcome of ear symptoms after COVID-19 infection - Tinnitus

□None □Completely disappeared after treatment

□Partially disappeared after treatment □Self-healing residual symptoms

1. Outcome of ear symptoms after COVID-19 infection - Ear fullness

□None □Completely disappeared after treatment

□Partially disappeared after treatment □Self-healing residual symptoms

1. Outcome of ear symptoms after COVID-19 infection - Ear pain

□None □Completely disappeared after treatment

□Partially disappeared after treatment □Self-healing residual symptoms

1. Outcome of ear symptoms after COVID-19 infection - Dizziness

□None □Completely disappeared after treatment

□Partially disappeared after treatment □Self-healing residual symptoms

**Cognitive and emotional state after COVID-19 infection**

1. Cognitive State after COVID-19 Infection:

□Able to remember most things, clear-minded, and able to solve daily problems.

□Able to remember most things, but have some difficulty thinking and solving daily problems.

□Somewhat forgetful, but clear-minded and able to solve daily problems.

□Somewhat forgetful and have some difficulty thinking and solving daily problems.

□Very forgetful and have great difficulty thinking and solving daily problems.

□Unable to remember anything and unable to think or solve daily problems.

1. Emotional State after COVID-19 Infection:

□Happy and interested in life.

□Somewhat happy.

□Somewhat unhappy.

□ Very unhappy.

□So unhappy to the point of feeling life has no value.

1. In the past two weeks, how often have you felt nervous, anxious, or restless?

□Not at all.

□A few days.

□More than half of the days.

□Almost every day.

1. In the past two weeks, how often have you been unable to stop or control worrying?

□Not at all.

□A few days.

□More than half of the days.

□Almost every day.

1. In the past two weeks, how often have you felt a lack of energy or interest in doing things?

□Not at all.

□A few days.

□More than half of the days.

□Almost every day.

1. In the past two weeks, how often have you felt down, depressed, or hopeless?

□Not at all.

□A few days.

□More than half of the days.

□Almost every day.

**Medical History**

1. Do you have ear, nose, and throat diseases? (such as tinnitus, otitis media, rhinitis, pharyngitis, etc.) If none, please write "none"____________
2. Do you have hypertension? (If yes, please write the duration of the disease, such as "5 years")

□Yes □No

1. Do you have diabetes? (If yes, please write the duration of the disease, such as "5 years")

□Yes □No
